# Sir James Paget, Bart., F. R. C. S., Eng., F. R. S.

**Published:** 1900-02

**Authors:** 


SIR JAMES PAGET, Bart., F. R. C. S., Eng., F. R. S., Sergeant-Surgeon to Her Majesty the Queen, and Consulting Surgeon to St. Bartholomew's Hospital, died in London, December 30, 1899, aged 85 years.

The life history of this great man is a most entertaining chapter in English medical annals. The story of his rise from obscurity in the little provincial town of Yarmouth, to renown in the great metropolis of the world is full of absorbing interest. It furnishes a lesson of encouragement to every young man, no matter how humble his origin, if he is but willing to work.

In his life he furnishd an example of industry, honesty, devotion to duty, courteous demeanor, sobriety, skill as a surgeon, eminence as a teacher, love of art, manliness, culture, all that goes to make a well-rounded character; in his death he leaves profound regret.

